# Lumbar Plexus Block Performance for Femur Fracture for a Hamamy Syndrome Patient

**DOI:** 10.5152/TJAR.2022.21095

**Published:** 2022-10-01

**Authors:** Yavuz Gürkan, İlayda Kalyoncu, Cemil Cihad Gedik, Mete Manici, Emel Gönen

**Affiliations:** 1 Department of Anaesthesiology, Koç University Faculty of Medicine, İstanbul, Turkey; 2 Department of Orthopaedic Surgery, Koç University Faculty of Medicine, İstanbul, Turkey

To the Editor,

Hamamy syndrome presents with disturbances in the development of the face, brain, bone, heart, and gonads, first described by Hamamy et al.^1^ Since the first description of the syndrome, there has been only a single case report on the anaesthetic management in this rare patient group.^2^

The Shamrock method^3^ for lumbar plexus block is a recent and effective method for providing analgesia in paediatric hip surgery patients.^4^

In this letter, we report the successful use of the Shamrock method for lumbar plexus block to provide postoperative analgesia for a patient with Hamamy syndrome who had intramedullary fixation for an occult femur fracture.

## Case Presentation

The patient was an American Society of Anesthesiologists Physical Status I (ASA I), 77 kg, 15-year-old boy scheduled to undergo intramedullary femoral nailing for an occult femur diaphysis fracture. Written informed consents from both the patient and his parents were obtained. The Hamamy syndrome diagnosis was made when he was 6.5 years old. In 2018, the patient underwent surgical treatment for a left proximal femur fracture at a different institution under general anaesthesia. The patient reported severe postoperative pain after his previous surgery and requested better pain management. We preferred lumbar plexus block for this patient because we wanted to avoid the potential side effects of the neuraxial blockade.

General anaesthesia was induced using 150 mg of propofol and remifentanil infusion. We avoided neuromuscular blockade during intubation to obtain response to nerve stimulation during block performance. The lumbar plexus block using the Shamrock method was performed in lateral decubitus position with ultrasound guidance using convex probe (GE Logiq S7 Ultrasound, GE Healthcare Korea, Seoul, Republic of Korea). Lumbar plexus block was performed using 20 mL of bupivacaine 0.25%. The nerve stimulator (Stimuplex HNS 12; B Braun Medical, Melsungen, Germany) was set to deliver 1.0 mA current impulses of 0.1 ms duration at a frequency of 2 Hz during nerve stimulation (NS). Local anaesthetic (LA) was administered only after obtaining a motor response of femoral nerve type at a current output of 0.5 mA.

Anaesthesia maintenance was provided using desflurane 1 minimum alveolar concentration (MAC) and remifentanil infusion as needed to maintain the surgical anaesthesia. At the end of surgery, patient received paracetamol 1000 mg/1 gr, tramadol 50 mg, and ibuprofen 400 mg to contribute to postoperative analgesia. Intravenous (IV) patient-controlled analgesia (PCA) device, using tramadol with 10 mg bolus dose and 20-minute lock-out duration, was available for rescue analgesic purposes.

Surgery lasted for 4 hours and 45 minutes, femoral osteotomy was added due to the bowing of the femur. During physical examination performed 12 hours after the surgery, the patient did not have any sensory or motor deficits. The patient’s pain values based on visual analog scale (VAS) from 1 to 10, were all zero for the first 6 hours. The patient had mild pain at 12th hour, with a VAS value of 3. The total tramadol consumption was 10, 30, and 40 mg at 6th, 12th, and 24th hours, respectively. The patient bed rested on the first postoperative day. On the second day, the patient was ambulatory, and his pain was managed with IV-PCA.

## Discussion

In this case, we preferred to use the Shamrock method to perform a lumbar plexus block due to its efficiency in providing analgesia for pediatric hip surgeries.^4^ The block provided sufficient analgesia to keep the patient pain-free during the first 24 hours and total analgesic consumption was only 40 mg of tramadol. Among the alternatives for this procedure could be epidural anaesthesia and caudal block, however they were not preferred due to their potential side effects related to central neuraxial blocks, such as urinary retention, pruritus, and hypotension.^5^ In cases where central neuraxial blocks are not preferred, lumbar plexus blocks seem a viable alternative in perioperative pain management in patients undergoing femoral nailing and osteotomy. To the the best of our knowledge, this is the first case report demonstrating the effective use of lumbar plexus block in Hamamy syndrome. We think that ultrasound-guided lumbar plexus blocks are quite effective in pediatric hip surgery and should be included in the armamentarium of anaesthesiologists.
